# Transcriptomic signature defines two subtypes of locally advanced PCa with distinct neoadjuvant therapy benefits

**DOI:** 10.3389/fonc.2023.963411

**Published:** 2023-05-17

**Authors:** Yinjie Zhu, Liancheng Fan, Hanjing Zhu, Yiming Gong, Chenfei Chi, Yanqing Wang, Jiahua Pan, Baijun Dong, Wei Xue

**Affiliations:** Department of Urology, Renji Hospital, School of Medicine, Shanghai Jiao Tong University, Shanghai, China

**Keywords:** predictive signature, locally advanced prostate cancer, transcriptomic profiling, neoadjuvant chemo-hormonal therapy, neoadjuvant hormonal therapy

## Abstract

**Background:**

Patients with locally advanced prostate cancer (LAPCa) received docetaxel-based neoadjuvant chemo-hormonal therapy (NCHT) had better clinical outcomes after surgery compared to neoadjuvant hormonal therapy (NHT) groups, but not all patients experienced favorable clinical outcomes with NCHT, raising the necessity for potential biomarker assessment. The transcriptomic profiling offers a unique opportunity to interrogate the accurate response to NCHT and NHT treatment and to identify the predictive biomarkers for neoadjuvant therapy.

**Methods:**

The whole transcriptomic profiling was performed on baseline biopsies and surgical tissue specimens from 64 patients with LAPCa at Renji Hospital between 2014 and 2018. Biochemical progression-free survival (bPFS)-based gene-by-treatment interaction effects were used to identify predictive biomarkers for guiding treatment selection.

**Results:**

Comparing the transcriptome profiling of pre- and post-treatment LAPCa specimens, NHT and NCHT shared 1917 up- and 670 down-regulated DEGs at least 2-fold. Pathway enrichment analysis showed up-regulated pathways in response to NHT and NCHT were both enriched in cytokine receptor interaction pathways, and down-regulated pathways in response to NCHT were enriched in cell cycle pathways. By comprehensive transcriptome profiling of 64 baseline specimens, ten predictive markers were identified. We integrated them into the signature to evaluate the relative benefits of neoadjuvant therapy, which categorizes patients into two subgroups with relative bPFS benefits from either NHCT or NHT. In the high-score (≥ -95.798) group (n = 37), NCHT treatment led to significantly longer bPFS (P< 0.0001), with a clear and early separation of the Kaplan–Meier curves. In the low-score (< -95.798) group (n = 27), NHT also led to significantly longer bPFS (P=0.0025).

**Conclusions:**

In this study, we proposed the first predictive transcriptomic signature might potentially guide the effective selection of neoadjuvant therapy in LAPCa and might provide precise guidance toward future personalized adjuvant therapy.

**Trial registration:**

The study was approved by the Ethics Committee of Renji Hospital affiliated to Shanghai Jiao Tong University (Ky2019-087).

## Implications for Practice:

The transcriptomic profiling offers a unique opportunity to interrogate the accurate response to NCHT and NHT treatment and to identify the predictive biomarkers for neoadjuvant therapy. Therefore, we proposed the first predictive transcriptomic signature could potentially guide the effective selection of neoadjuvant therapy in patients with locally advanced prostate cancer in the Chinese population and might provide precise guidance toward future personalized adjuvant therapy.

## Introduction

Prostate cancer (PCa) remains one of the most commonly diagnosed malignancies worldwide (1). Nowadays, major shifts in PCa incidence in China might be related to widespread prostate-specific antigen (PSA) screening, and the popular Western-style diets (2). Up till now, the majority of PCa patients were still diagnosed at the advanced stage in China (3). For the locally advanced PCa (LAPCa) patients, evidence-based guidelines recommend radical prostatectomy (RP) as the primary treatment and most of them will benefit from this procedure (4–6). However, due to the large tumor load in LAPCa patients, RP alone poses a great challenge in post-operative tumor control and surgical difficulties. Therefore, personalized neoadjuvant therapy is particularly important. Currently, the optimal comprehensive neoadjuvant therapy for LAPCa patients is still controversial.

Neoadjuvant hormonal therapy (NHT) before RP for LAPCa has shown promises in local disease control, but not in survival outcomes in randomized clinical trials (7). Neoadjuvant chemohormonal therapy (NCHT) before RP can also reduce the risk of biochemical recurrence (BCR) in patients with LAPCa (8, 9). A real-world data analysis of our center suggested that NCHT before RP significantly improved patient outcomes compared with NHT and RP alone without neoadjuvant therapy (10). Although the overall prognosis of NHCT is excellent, there is still significant heterogeneity in the clinical benefit of those patients. To date, very few studies have addressed the suitable biomarkers to help physicians prioritize the application of NHT versus NHCT for patients with LAPCa.

With the progression of high throughput gene-sequencing technology, understanding of tumor genome and the development of tumor molecular typing has been greatly promoted. RNA sequencing (RNA-seq) contributes to the accurate classification of PCa to guide the selection of treatment options and helps to explore the molecular mechanism of drug resistance in PCa (11, 12). However, current studies on the accurate classification of PCa based on gene expression profiles mainly focus on the Caucasian population, and there is still a lack of data in the Chinese population. In addition, the specific pharmacodynamic mechanism of these treatments has not yet been reported, which greatly limits the clinical applications of neoadjuvant treatment. Several studies have shown that the occurrence, development, and migration of cancer cells could be closely related to the metabolic reprogramming of tumor cells (13). We also aimed at understanding the clinical efficacy of docetaxel in the neoadjuvant treatment by analyzing the transcriptomic landscape of pre-and post-treatment tumor tissues in LAPCa.

In this study, we conducted a comparative study on patients with LAPCa who received NCHT or NHT before RP. We compared the transcriptomic landscape of NHT and NCHT to investigate the gene expression changes in patients with LAPCa in response to docetaxel in the neoadjuvant treatment. Then we further screened the predictive markers from baseline RNA-seq data and built the predictive signature to guide the optimum selection of the neoadjuvant treatment.

## Patients and method

### Patient selection and grouping

From 2014 to 2018, a total of 338 patients whom had LAPCa intended to receive neoadjuvant therapy and RP in Renji Hospital. Patients were enrolled according to the following criteria (1): male, age 18-75; (ii) Based on EAU-ESTRO-SIOG 2015 guideline, patients were initially diagnosed as LAPCa and could be treated with RP and ePLND; (iii) patients had a good general performance status with ECOG score 0-1;(iv) adequate haematological, hepatic, and renal function.

**Figure 1:**
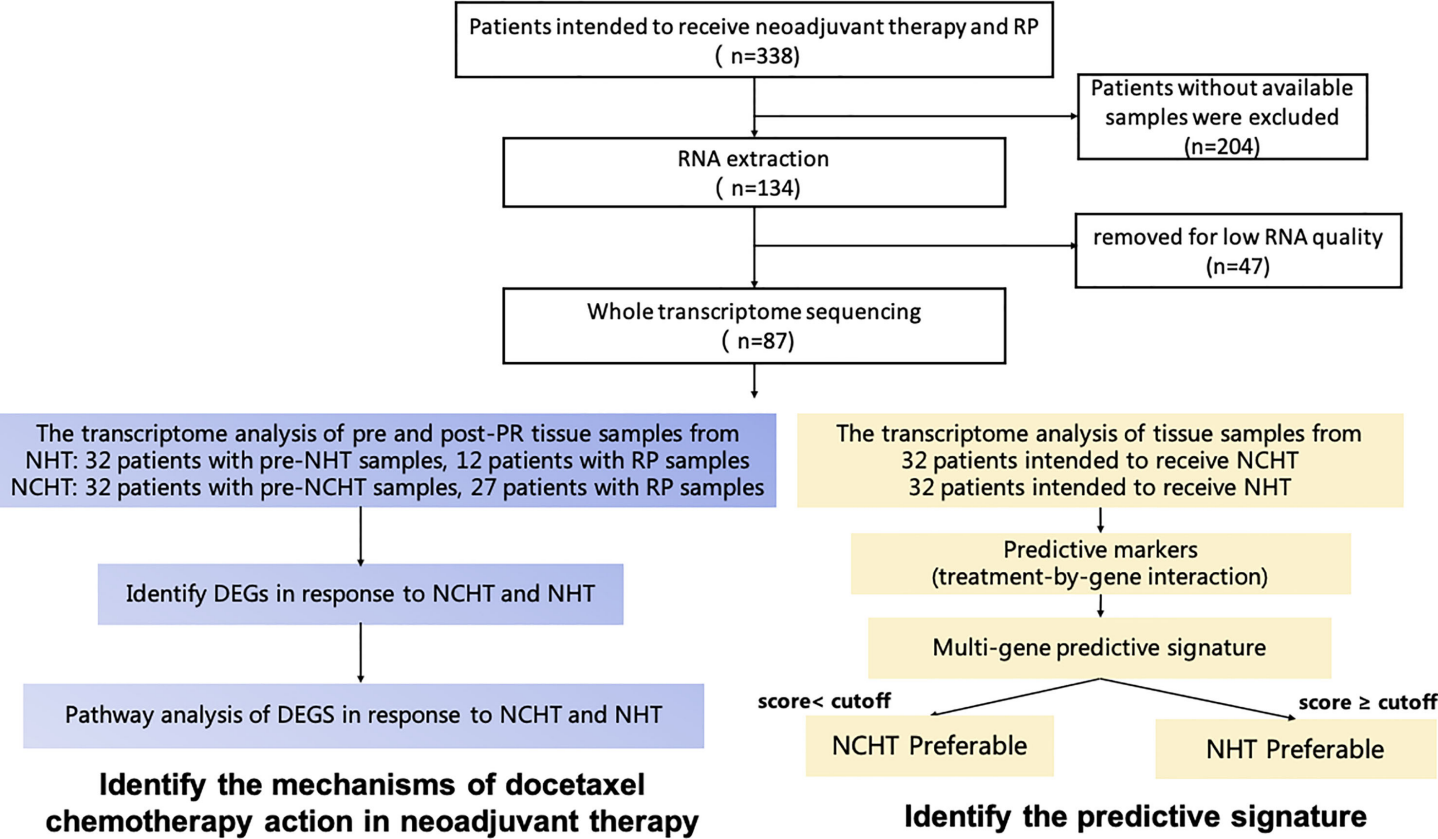
Schematic diagram of sample collection, and methodology for developing the clinical predictive model and identifying mechanisms of docetaxel chemotherapy action in neoadjuvant therapy.

Tissue samples were obtained from patients with LAPCa who underwent RP. Biopsy samples were obtained from patients with LAPCa before the neoadjuvant therapy. The analysis process of this study was depicted in **Figure 1**. According to the decision based on the surgeon’s suggestion and patients’ choices, patients received NCHT or NHT preoperative management. Informed consent was provided by all the patients, and the study was approved by the Ethics Committee of Renji Hospital affiliated with Shanghai Jiao Tong University (Ky2019-087).

### Neoadjuvant treatment and radical prostatectomy

For patients in the NCHT group, 4 to 6 cycles of androgen deprivation therapy (ADT) and docetaxel-based systemic chemotherapy were administrated. Docetaxel was administrated intravenously at a dose of 75 mg/m^2^ every 3 weeks along with oral administration of prednisone 5 mg twice a day. RP was performed 3-4 weeks after the neoadjuvant therapy by 2 experienced surgeons.

### RNA extraction, library preparation, and sequencing

The RNA samples were isolated and sent to the Mingma technologies (Shanghai, China) for constructing the RNA-seq library. Briefly, total RNA was extracted by the RNeasy Mini Kit (QIAgen, 74104). RNA quantity and quality, with the calculation of RNA integrity number (RIN), were accessed by The NanoDrop 2000 spectrophotometer (Thermo Scientific) and 2100 Bioanalyzer (Agilent). Poor samples were excluded by RIN< 8 or total RNA< 1.1 ug. Samples were selected for strand-specific total RNA library preparation in a TruSeq Stranded Total RNA Gold library protocol (Illumina, San Diego, CA), which is recommended for low input amounts or degraded RNA samples (14). After normalization, the libraries were sequenced in 4-plex on an Illumina Novaseq 6000 system to create paired-end reads with a length of 150 bp (PE150) (Illumina, San Diego, CA, USA).

### Selection of predictive gene features

We followed the popular approach to select predictive genes and develop the predictive signature (15–17). We first performed an interaction test between treatment and each candidate gene separately. For a particular gene feature, we assume a standard multivariate Cox proportional hazards model:


$$h_{i}(t)=h_{0}(t)exp\{\beta_{1}t_{i}+\beta_{2}g_{i}+\beta_{3}t_{i}g_{i}\}$$

Where *h_0_ (t)* is the baseline hazard and *β* are the regression parameters, *gi* is the gene expression level, *t_i_* denotes the treatment assignment for each patient *_i_*, such that *t_i_* = 0 indicates that the patient received NCHT and *t_i_* = 1 indicates that the patient received NHT, and the product *t_i_g_i_* represents the interaction term of treatment and the gene expression level.

The selection of biomarker is based on the Wald test statistic for testing a null interaction effect, *β*_3 =_ 0. _A_ standardized test statistic, z, that approximately follows the standard normal distribution under the null interaction effect was calculated by:


$$z=\frac{\beta_{3}}{se(\beta_{3})}$$

Here, z is the standard Wald statistic of the multi-factor Cox proportional hazards model without interaction (*β*_3_=0). After standardization, a negative or positive z value represents that high gene expression is associated with better outcomes with NHT or NCHT, respectively. A set of features with a significance level of this test statistic less than 0.05 were selected to generate the multi-gene signature.

### Development of the predictive signature

Based on interaction z-scores (also called differential neoadjuvant treatment predictive score [DNTP score]) of the ten predictive markers, we could calculate the score using the following function:


$$\begin{array}{l} score=3.74*GNAS+(-3.65)*COX15+(-3.693)*NMRK1+\\ (-4.332)*CLYBL+(-3.629)*PNCK+\\ (-3.75)*MMS19+(-3.514)*COL4A5+(-3.569)*ZNF774+\\ (-3.654)*HBA1+(-3.496)*DBNDD2 \end{array}$$

Smaller values of score correspond to a greater chance of benefiting from NHT than from NCHT. Based on the combination of predictive genes of each score, we screened the different cutoffs of the predictive signature and chose to categorize the patients into two subgroups with scores of<−95.798 and ≥−95.798 with distinct biochemical progression-free survival (bPFS) differences.

### Bioinformatic analysis

Genes with DEseq2 |logFC|>1 and adjust-pval<0.05 were identified as differentially expressed genes (DEGs). For $\log_{2}^{\left(\text{TPM}+1\right)}$ expression data, the R package of “removeBatchEffect” was used to remove the batch effect and also kept the treatment information. Gene set enrichment analysis (GSEA) and Kyoto Encyclopedia of Genes and Genomes (KEGG) were analyzed by the “clusterProfiler” R package (v3.18.1), based on MSigDB gene set “c2.cp.kegg.v7.4.entrez.gmt”. P-values adjusted by the Benjamini-Hochberg method (adj.p-value< 0.05) were employed to select statistically significant KEGG terms. Principal Component Analysis (PCA) was carried out using the R package ‘princomp’ to analyze the expression pattern of grouped patients.

The significantly enriched pathways and the detailed results were presented in **Supplementary Tables 1**, **2**, where the “geneID” column represented the specific genes enriched in each pathway. In addition, regarding the up- and down-regulation of specific genes in each pathway under the two treatment methods, we conducted the further analysis. The gene lists enriched in each pathway can be found in **Supplementary Table 3**. The Jaccard similarity coefficient was used to measure the consistency of up-regulated genes in the pathways between the two treatment methods. It calculates the ratio between the intersection and union of two sets, as shown in the following formula:


J(A,B)=|A∩B|/|A∪B|

Here, A and B represent the two gene lists, and |A∩B| and |A∪B| represent the intersection and union of the two gene lists, respectively. The Jaccard similarity coefficient ranges from 0 to 1, with a value closer to 1 indicating a higher similarity between the two gene lists. The results of the calculation can be found in **Supplementary Table 3**, where “level1_consis” represents the number of genes significantly up-regulated under both treatment methods, and “level2_consis” represents the number of genes significantly up-regulated under one treatment method but still up-regulated (albeit not significantly) under the other treatment method. Based on this, we defined the median Jaccard similarity coefficient of level 1 genes (column “level 1_coef”) to be close to 0.7, with the highest value reaching 0.96. After including level 2 genes, the median Jaccard similarity coefficient (“consis_coef”) was 1, indicating a high consistency in the up-regulation of genes not only at the pathway level, but also at the gene expression level between NHT and NCHT treatments.

The original enrichment results of down-regulated pathways after the two treatments can be found in **Supplementary Tables 4**, **5**, and the consistency results at the gene level can be found in **Supplementary Table 6**. The oocyte meiosis and cell cycle pathways showed low consistency between the two treatment methods, which is consistent with the two pathways being primarily down-regulated in a treatment-specific manner by NCHT. In contrast, other down-regulated pathways showed higher consistency between the two treatment methods.

### Statistical analysis

Univariate and multivariate analyses of the association of biomarkers, treatment interaction, and clinical factors with bPFS were performed with the Cox proportional hazard regression model. Means and differences of the means with a 95% confidence interval (CI) were calculated using Wilson’s score CI. A p-value of<0.05 was considered statistically significant. Differences between the groups were calculated using the Wilcox test, or the Fisher’s exact test, where appropriate. Kaplan–Meier curves of bPFS were estimated for each subgroup, and statistically compared using the log-rank test. A log rank test P-value< 0.5 was considered statistically significant. All statistical analyses were performed with the R statistics package (R version 3.4.0; R: The R-Project for Statistical Computing, Vienna, Austria).

## Result

### Baseline clinical and pathological features

Total of 64 LAPCa patients with available baseline biopsy specimens have been enrolled for transcriptomic profiling (**Figure 1**). There were 32 patients in the NCHT treatment group, and 32 patients in the NHT treatment group. As shown in **Table 1**, The NCHT group had a significantly higher initial baseline prostate-specific antigen (PSA) level, than the NHT groups (P=0.04). Other clinicopathological factors were well balanced between the two groups. Moreover, the proportion of patients with clinical node-positive disease was 62.5% and 37.50% in the NCHT group and NHT group, respectively. After 4 to 6 cycles of neoadjuvant therapy, the rate of biochemical recurrence was 53.12% and 62.50%, respectively.

**Table 1:** Clinicopathological data of the patients in NHCT and NCHT groups.

	Overall (n = 64)	NCHT (n = 32)	NHT (n = 32)	p
Age				
Mean(SD)	67.88 (5.44)	66.97 (5.68)	68.78 (5.12)	0.144 (wilcox.test)
median[IQR]	68.00 [64.00, 71.25]	67.00 [63.00, 70.25]	69.00 [66.00, 72.25]	
Initial PSA				
0-10 ng/ml	2 (3.12%)	1 (3.12%)	1 (3.12%)	0.040 (fisher.test)
10-20 ng/ml	2 (3.12%)	1 (3.12%)	1 (3.12%)	
20-100 ng/ml	32 (50.00%)	11 (34.38%)	21 (65.62%)	
>100 ng/ml	28 (43.75%)	19 (59.38%)	9 (28.12%)	
Preoperative PSA				
0-10 ng/ml	59 (92.19%)	32 (100.00%)	27 (84.38%)	0.053 (fisher.test)
10-20 ng/ml	3 (4.69%)	0 (0.00%)	3 (9.38%)	
20-100 ng/ml	1 (1.56%)	0 (0.00%)	1 (3.12%)	
Missing	1 (1.56%)	0 (0.00%)	1 (3.12%)	
Gleason score				
< 8	23 (35.94%)	12 (37.50%)	11 (34.38%)	1.000 (fisher.test)
≥ 8	41 (64.06%)	20 (62.50%)	21 (65.62%)	
T stage				
T2c	3 (4.69%)	1 (3.12%)	2 (6.25%)	0.210 (fisher.test)
T3a	21 (32.81%)	7 (21.88%)	14 (43.75%)	
T3b	21 (32.81%)	13 (40.62%)	8 (25.00%)	
T4	19 (29.69%)	11 (34.38%)	8 (25.00%)	
N stage				
N0	32 (50.00%)	12 (37.50%)	20 (62.50%)	0.079 (fisher.test)
N1	32 (50.00%)	20 (62.50%)	12 (37.50%)	
Biochemical recurrence				
Yes	37 (57.81%)	17 (53.12%)	20 (62.50%)	0.613 (fisher.test)
No	27 (42.19%)	15 (46.88%)	12 (37.50%)	

### The transcriptomic changes of locally advanced PCa in response to the neoadjuvant treatment.

**Figure 2:**
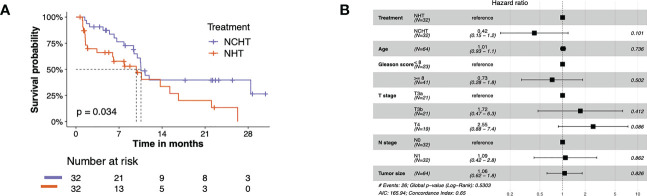
The bPFS survival profiles of patients in NCHT and NHT groups. **(A)** Kaplan–Meier curves estimate the bPFS of the pre-categorized cohort which received neoadjuvant NHT or NCHT (N = 64). **(B)** Forest plot of risk factors affecting the bPFS in PCa.

The bPFS survival profiles of patients in NCHT and NHT groups were shown in **Figure 2A**. The NCHT group had a longer bPFS after surgery compared to the NHT group (p=0.034; HR 2.02 [1.04-3.91]). The median bPFS of the NCHT group and NHT group were 10.57 and 9.8 months, respectively. However, there were still patients who cannot benefit from the NCHT, 28% (9 out of 32) of NCHT patients experienced fast progression (bPFS< 6 months). In addition, **Figure 2B** also indicated that treatment (NCHT or NHT) was not an independent prognostic indicator by multivariate Cox regression analysis. It is difficult to determine the optimal treatment for primary PCa. Therefore, we need predictive biomarkers to distinguish the patients who might benefit from NCHT and the patients who benefit from NHT.

RNA-seq was performed on 64 baseline PCa lesions and 39 post-NHT and NCHT PCa specimens. First, we performed dimension reduction on the baseline samples using PCA. The results showed that baseline samples from NCHT and NHT groups cannot be clearly separated into two major dimensions (principal components 1 and 2, **Supplementary Figure 1A**). Only three downregulated DEGs were identified at baseline, including the well-established ETS-related gene (*ERG)* (**Supplementary Figure 1B**).

**Figure 3:**
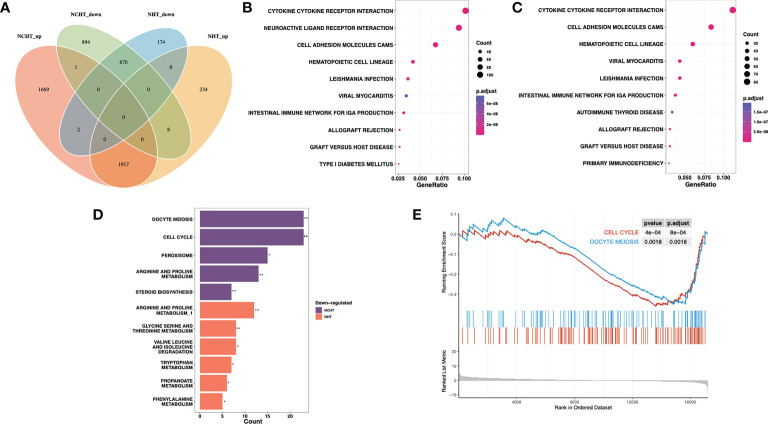
Differentially-expressed genes and pathways following NHT and NHCT. **(A)** Venn diagram of up-regulated and down-regulated DEGs in response to NHT and NCHT. **(B, C)** Dotplots showing enrichment of KEGG pathways among post-NCHT and NHT upregulated genes. **(D)** Barplot showing KEGG pathways enriched in post-NCHT and NHT down-regulated genes **(E)** Gene set enrichment analysis (GSEA) found that NHCT mainly exhibited enrichment of the cell cycle and oocyte meiosis pathways. ** P<0.01, *P<0.01.

We further addressed the transcriptomic landscape of NHT and NCHT in LAPCa. Comparing the gene expression profiles of pre-and post-treatment LAPCa specimens, NHT and NCHT shared 1917 up-regulated DEGs, and 670 down-regulated DEGs at least 2-fold (**Figure 3A**). To identify biological pathways perturbed by NHT and NCHT, we performed functional analysis on DEGs with KEGG function analysis. The biological process of KEGG pathway enrichment showed upregulated pathways after NHT and NCHT were similar and mainly associated with cytokine receptor interaction, cell adhesion molecular pathways etc. (**Figures 3B, C**). The KEGG terms “cell cycle” and “oocyte meiosis” were enriched in the down-regulated gene list in response to NCHT (**Figure 3D** and **Supplementary Figure 2**). Stratified gene set enrichment analysis (GSEA) pathway analysis also confirmed that cell cycle and oocyte meiosis-related pathways were enriched (**Figure 3E**). Additionally, we observed the high consistency of up- and down-regulated genes in the pathways between the two treatment methods (**Supplementary Tables 1**-**6**). All of the above results implicated that NCHT may suppress the cell cycle and oocyte meiosis-related pathways in LAPCa.

### Identification of predictive biomarkers for guiding treatment selection

**Figure 4:**
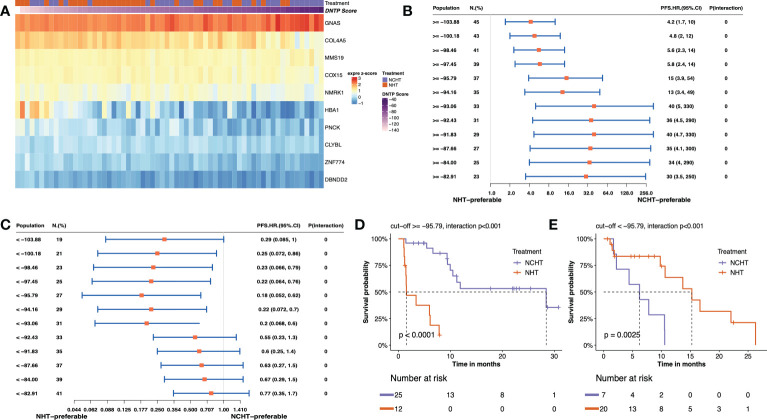
Predictive value of transcriptomic signature for bPFS in each subgroup. **(A)** Heatmap of z-score of 10 predictive markers. **(B, C)** Forest plot showing the treatment-by-interaction hazard ratio (HR) and interaction p-value of bPFS with the Cox regression model as classified by cutoff scores. **(D, E)**. Kaplan–Meier curves of bPFS for patients treated by neoadjuvant NHCT or NHT in two subgroups.

In this study, we adopted the popular approach of testing bPFS-based gene-by-treatment interaction effects to identify predictive biomarkers for guiding treatment selection (17–19). We evaluated the predictive power of each marker and finally identified 10 genes with significant treatment interaction (**Table 2**, **Figure 4A**, method section). Here, the negative z-score indicated relatively better survival with NHT while the positive z-score indicated relative benefit with NCHT. Under the gene-by-treatment test, *GNAS* (z-score, 3.74) and *CLYBL* (z-score, -4.332) were the strongest predictors for neoadjuvant therapies but in opposite directions. Here, the high expression of *GNAS* indicated relative benefit with NCHT, while the high expression of *CLYBL* indicated relative benefit with NHT.

**Table 2:** Predictive values of 10 identified markers (treatment-by-gene interaction).

Gene	iHR (95% CI)	interaction p	z-score	favor
*GNAS*	114.162 (9.535-1366.872)	<0.001	3.74	NCHT
*COX15*	0.019 (0.002-0.159)	<0.001	-3.65	NHT
*NMRK1*	0.073 (0.018-0.293)	<0.001	-3.693	NHT
*CLYBL*	0.031 (0.007-0.15)	<0.001	-4.332	NHT
*PNCK*	0.293 (0.151-0.568)	<0.001	-3.629	NHT
*MMS19*	0.03 (0.005-0.188)	<0.001	-3.75	NHT
*COL4A5*	0.149 (0.051-0.43)	<0.001	-3.514	NHT
*ZNF774*	0.155 (0.056-0.432)	<0.001	-3.569	NHT
*HBA1*	0.523 (0.37-0.741)	<0.001	-3.654	NHT
*DBNDD2*	0.225 (0.097-0.519)	<0.001	-3.496	NHT

The interaction hazard ratio (iHR) is the effect value brought by the interaction item.

We then constructed a predictive signature to quantitatively assess individual tumors and their corresponding treatment responses by weighting z scores from individual treatment-by-interaction tests of those selected genes. Further, to stratify patients for particular treatment benefits, we screened the different cutoff values of the predictive signature and chose to categorize the patients into two subgroups at -95.79 as they resulted in the best bPFS differences (**Figures 4B, C** and **Supplementary Figure 3**). Both high-score (≥ -95.79) and low-score (<-95.79) patients manifested a more pronounced bPFS difference between NHT and NCHT. The inter-treatment difference for PFS reached statistical significance among high-score and low core patients (interaction term p<0.001). Remarkably, after categorization by cutoff score, the two subgroups demonstrated distinct treatment responses and the stratification of bPFS. In the high-score subgroup (≥ -95.79, n = 37), NCHT treatment led to a significantly longer bPFS (P< 0.0001) with a clear and early separation of the Kaplan–Meier curves (**Figure 4D**). In the low-score subgroup (< -95.79 group, n = 27), NHT also led to a significantly longer bPFS (P=0.0025) with a clear and early separation of the Kaplan–Meier curves (**Figure 4E**). After grouping by the threshold, the high-score group did benefit significantly from NCHT treatment, while the low-score group benefited significantly from NHT treatment, and there was a significant interaction between grouping and treatment.

**Figure 5:**
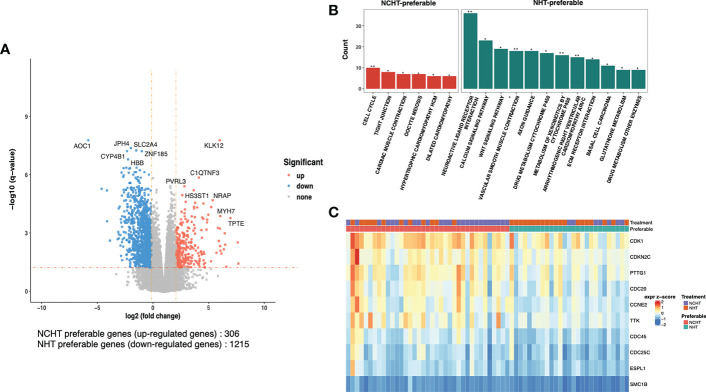
Differentially-expressed genes and pathways in NCHT-preferable and NHT-preferable groups. **(A)** Volcano plot of the transcriptomic landscape of NCHT-preferable and NHT-preferable groups (DEseq2 |logFC|>2 and adjust-p val<0.05). **(B)** Barplots showing enrichment of pathways in NCHT-preferable and NHT-preferable genes. **(C)** Heatmap of z-score of the key positive cell cycle regulators. ** P<0.01, *P<0.01.

To understand the underlying molecular difference between NCHT-preferable and NHT-preferable patients, we analyzed the baseline gene expression profiles of NCHT-preferable and NHT-preferable patients determined by our predictive signature. Remarkably, clinicopathological factors were well balanced between NCHT-preferable and NHT-preferable groups (**Table 3**). 306 NCHT-preferable genes and 1215 NCHT-preferable genes were identified at baseline (**Figure 5A**). NCHT preferable group were significantly enriched in genes within the KEGG term “Cell Cycle” including the key positive cell cycle regulators *CDK1*, *CDKN2C*, *PTTG1* and *CDC20* (**Figures 5B, C**). After categorization by the predictive signature, the two subgroups demonstrated distinct treatment responses and underlying molecular profiles, but similar clinicopathological features.

**Table 3:** Clinicopathological data of the patients in NCHT-preferable and NHT-preferable groups.

	Overall (n = 64)	NCHT-preferable (n = 37)	NHT-preferable (n = 27)	p
Treatment				
NCHT	32 (50.00%)	25 (67.57%)	7 (25.93%)	0.002 (fisher.test)
NHT	32 (50.00%)	12 (32.43%)	20 (74.07%)	
Age				
Mean(SD)	67.88 (5.44)	67.27 (5.85)	68.7 (4.81)	0.337 (wilcox.test)
median[IQR]	68.00 [64.00, 71.25]	67.00 [63.00, 71.00]	68.00 [66.00, 72.50]	
Initial PSA				
0-10 ng/ml	2 (3.12%)	1 (2.70%)	1 (3.70%)	0.513 (fisher.test)
10-20 ng/ml	2 (3.12%)	2 (5.41%)	0 (0.00%)	
20-100 ng/ml	32 (50.00%)	16 (43.24%)	16 (59.26%)	
>100 ng/ml	28 (43.75%)	18 (48.65%)	10 (37.04%)	
Preoperative PSA				
0-10 ng/ml	59 (92.19%)	35 (94.59%)	24 (88.89%)	0.386 (fisher.test)
10-20 ng/ml	3 (4.69%)	1 (2.70%)	2 (7.41%)	
20-100 ng/ml	1 (1.56%)	0 (0.00%)	1 (3.70%)	
Missing	1 (1.56%)	1 (2.70%)	0 (0.00%)	
Postoperative PSA				
0-10 ng/ml	59 (92.19%)	35 (94.59%)	24 (88.89%)	0.515 (fisher.test)
20-100 ng/ml	2 (3.12%)	2 (5.41%)	0 (0.00%)	
Missing	3 (4.69%)	0 (0.00%)	3 (11.11%)	
Gleason score				
< 8	23 (35.94%)	16 (43.24%)	7 (25.93%)	0.192 (fisher.test)
≥ 8	41 (64.06%)	21 (56.76%)	20 (74.07%)	
T stage				
T2c	3 (4.69%)	1 (2.70%)	2 (7.41%)	0.761 (fisher.test)
T3a	21 (32.81%)	11 (29.73%)	10 (37.04%)	
T3b	21 (32.81%)	13 (35.14%)	8 (29.63%)	
T4	19 (29.69%)	12 (32.43%)	7 (25.93%)	
N stage				
N0	32 (50.00%)	16 (43.24%)	16 (59.26%)	0.311 (fisher.test)
N1	32 (50.00%)	21 (56.76%)	11 (40.74%)	

## Discussion

Recently, there was a paradigm shift in the management of LAPCa. Several studies, including our previous study, have presented the introduction of early docetaxel chemotherapy in addition to ADT resulted in significantly better clinical outcomes than with standard ADT in LAPCa (20). Although docetaxel with ADT combination treatment could improve patient survival, some tumors are still chemo-resistant. It suggested the heterogeneous nature of PCa, necessitating effective predictive biomarkers to help determine which neoadjuvant therapy is warranted before patients undergo RP.

In this study, we analyzed the whole transcriptomic landscape of NHT and NCHT in LAPCa. In this biomarker exploration of neoadjuvant therapy, we selected the genes that could predict the relative benefit between NHCT and NHT and then integrated these biomarkers into a predictive signature. Notably, two groups separated by a cutoff score leaded significant bPFS segregation. In high-score group, patients showed better overall survival with NHT than with NCHT. We also found patients with activated genes involved in the cell cycle are more responsive to NCHT treatment. Our predictive signature for neoadjuvant therapy could potentially identify LAPCa patients who are more likely to benefit from one treatment over another treatment, providing clearer value to guide personalized neoadjuvant therapy.

At present, there have been many attempts to conduct a comprehensive molecular classification of PCa, and further study of the heterogeneity of PCa will provide a more in-depth understanding of the tumor genome and provide personalized neoadjuvant therapy. ERG expression may have a potential predictive value with respect to the effectiveness and outcome of docetaxel chemotherapy combined with an ADT regimen in metastatic hormone-sensitive PCa (21). Carcinomas originate from epithelial tissues and can be grouped into Luminal and Basal, and subtypes by the PAM50 subtyping (22). Luminal subtypes have been shown to express higher levels of hormone receptors and respond better to hormonal therapy in hormone-driven pan-cancer (11, 22, 23). The luminal subtypes of PCa exhibited higher expression of AR and luminal B-like tumors preferentially benefited from ADT (11). Through gene expression profiling, Anis Hamid et al. further analyzed luminal-basal molecular typing in the CHAARTED study and evaluated its association with prognosis by stratification (12). Luminal B subtype is associated with poorer OS with ADT alone and benefits from the addition of docetaxel. In contrast, the Basal subtype shows a lack of OS benefit from NHCT combination therapy. However, recent studies on predictive markers for neoadjuvant therapy are mainly focused on the European and American populations, and there is still a lack of studies on the Chinese population. To the best of our knowledge, our predictive signature provides the first attempt at development of a multi-gene clinical predictive signature in the Chinese population, thus suggesting the predictive value of guiding personalized neoadjuvant therapy in LAPCa patients in China.

It was widely recognized that chemotherapy in neoadjuvant therapy may improve survival because of the existence and emergence of hormonally resistant cellular clones during the ADT treatment (24). In our study, we found NCHT showed a general reduction of cell cycle progression and cell cycle-related positive regulators, which is agreed with the previous study (25). The occurrence, development, and metastasis of tumors are closely related to the cell cycle. Our results suggest a persistent anti-tumourigenic effect of docetaxel chemotherapy action in neoadjuvant therapy.

Comprehensive characterization of the value of our study suffers several limitations. Due to the retrospective design and relatively small sample size, the possibility of selection bias could not be excluded. Development of this transcriptomic signature was based on a relatively small training cohort, which may introduce biased biomarker selection or an overfitted model. In addition, there were no equivalent public or clinical datasets of neoadjuvant therapy-treated patients with regular follow-up of survival outcomes available at the time of this study. Well-designed prospective validation studies are warranted to evaluate the predictive value of this signature in the future.

Taken together, we identified ten predictive markers for guiding neoadjuvant therapy and incorporated them into a multi-gene predictive signature to aid the neoadjuvant paradigm in PCa. Our preliminary multi-gene signature is the first predictive transcriptomic signature for guiding personalized and advanced neoadjuvant therapies. A future prospective study is warranted to evaluate the clinical value of this signature. In addition, we also demonstrated that NCHT appears to exhibit an expected mechanism of action on cell cycle progression.

## Data availability statement

The original contributions presented in the study are included in the article/**Supplementary Material**. Further inquiries can be directed to the corresponding authors.

## Ethics statement

The studies involving human participants were reviewed and approved by Renji Hospital affiliated with Shanghai Jiao Tong University (Ky2019-087). The patients/participants provided their written informed consent to participate in this study.

## Author contributions

BD and WX designed research; YZ, LF, HZ and YG performed research; CC, YW, and JP contributed new reagents/analytic tools; YZ and LF wrote the paper. All authors contributed to the article and approved the submitted version.
