# Supplementary material for: Transcriptomic signature defines two subtypes of locally advanced PCa with distinct neoadjuvant therapy benefits
Source: Front Oncol. 2023 May 17;13:963411. doi: 10.3389/fonc.2023.963411 (PMC10229793; doi:10.3389/fonc.2023.963411)
Supplement: Supplementary file 1 [file DataSheet_1.pdf]

Supplementary Figure 1: Baseline gene expression profiles in NCHT and NHT groups.

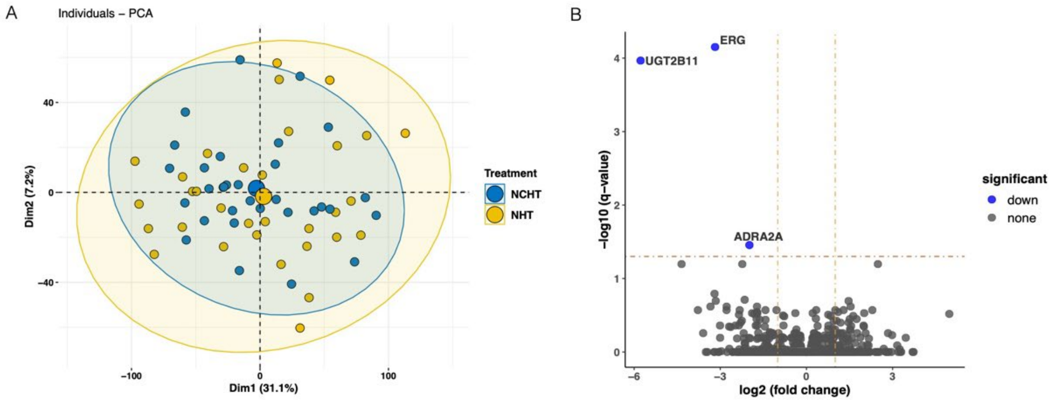

A. Principal component analysis (PCA) plot based on all genes of the study. B. Volcano plot of gene expression profiles of NHT and NCHT at baseline.

Supplementary Figure 2: Differences in transcriptome profiles pre- and post-neoadjuvant treatments.

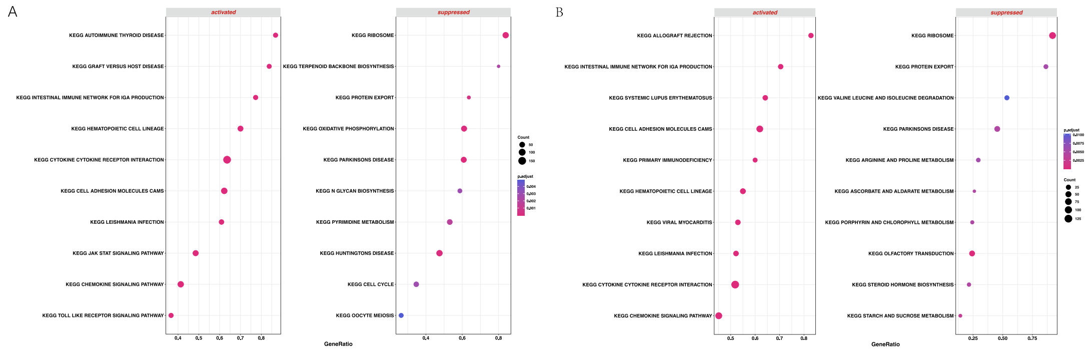

Bubble charts showing Kyoto Encyclopedia of Genes and Genomes (KEGG) pathways that activated (A) and suppressed (B) in the NCHT were enriched. Bubble chart showing KEGG pathways that activated (C) and suppressed (D) in the NHT were enriched.

Supplementary Figure 3: Forest plot showing the treatment-by-interaction hazard ratio (HR) and interaction p-value of bPFS with the Cox regression model as classified by cutoff scores.

A.

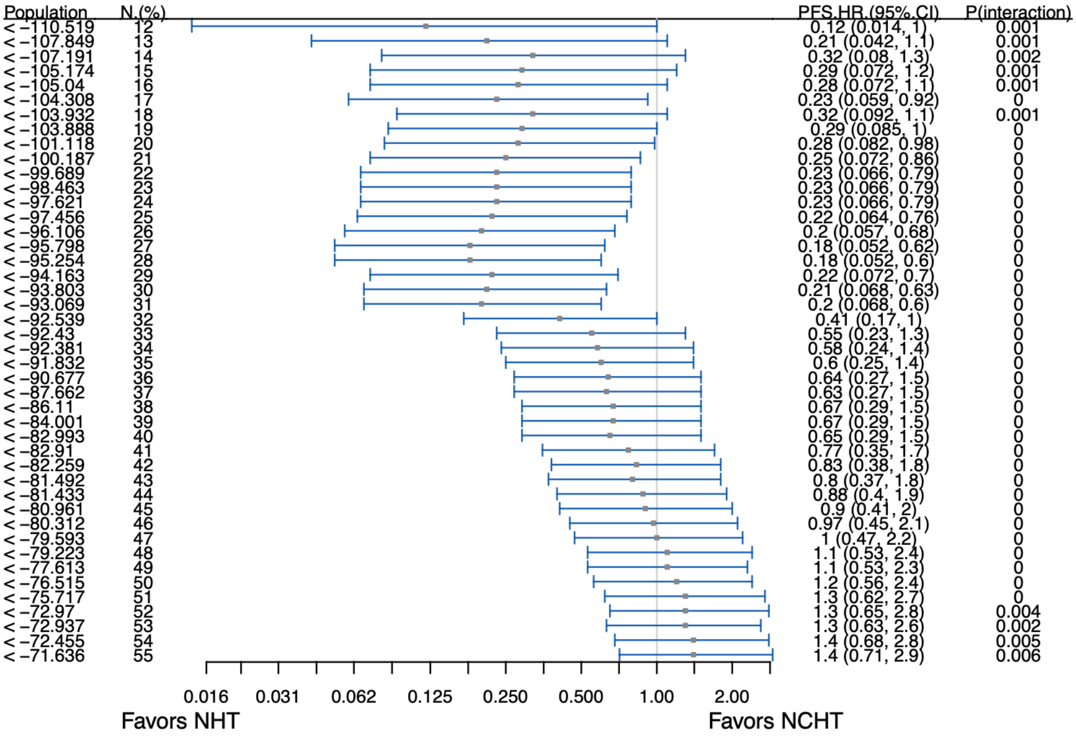

B.

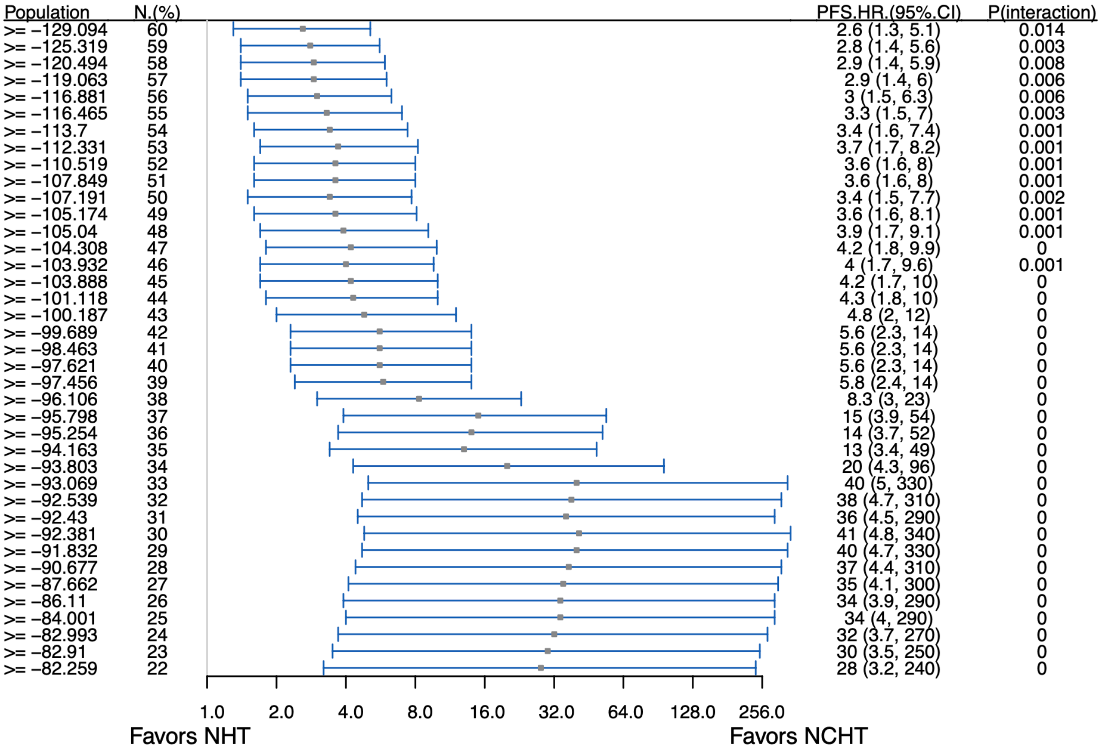

(A) Threshold selection diagrams from low to high (B) Threshold selection diagrams from high to low.
